# Walking Ability Outcome Measures in Individuals with Spinal Cord Injury: A Systematic Review

**DOI:** 10.3390/ijerph18189517

**Published:** 2021-09-09

**Authors:** Isabel Sinovas-Alonso, Ángel Gil-Agudo, Roberto Cano-de-la-Cuerda, Antonio J. del-Ama

**Affiliations:** 1 International Doctoral School, Rey Juan Carlos University, 28008 Madrid, Spain; 2 Biomechanics and Technical Aids Unit, Hospital Nacional de Parapléjicos, 45071 Toledo, Spain; amgila@sescam.jccm.es (Á.G.-A.); antonio.delama@urjc.es (A.J.d.-A.); 3 Department of Physical Therapy, Occupational Therapy, Physical Medicine and Rehabilitation, Faculty of Health Sciences, Rey Juan Carlos University, Alcorcón, 28922 Madrid, Spain; 4 Department of Applied Mathematics, Materials Science and Engineering and Electronic Technology, School of Science and Technology, Rey Juan Carlos University, Móstoles, 28933 Madrid, Spain

**Keywords:** spinal cord injury, walking ability, outcome measures, psychometric properties

## Abstract

Walking function recovery in spinal cord injury (SCI) is tackled through several therapeutic approaches in which precise evaluation is essential. A systematic review was performed to provide an updated qualitative review of walking ability outcome measures in SCI and to analyze their psychometric properties. PubMed, Cochrane, and PEDro databases were consulted until 1 April 2020. Seventeen articles written in English were included. Five of them studied the walking index for SCI, four studied the 10 meter walk test, and two studied the six-minute walk test, the timed Up and go test, and the Berg balance scale. The rest of the articles studied the following metrics: gait profile score, spinal cord injury functional ambulation profile, five times sit-to-stand test, spinal cord injury functional ambulation inventory, spinal cord independence measure (indoors and outdoors mobility items), locomotor stages in spinal cord injury, community balance and mobility scale, and activity-based balance level evaluation scale. The choice of a single or a set of metrics should be determined by the clinician. Based on the results obtained in this review, a combination of outcome measures is proposed to assess walking ability. Future work is required to integrate a more realistic environment for walking assessment.

## 1. Introduction

Annually, an estimated 250,000–500,000 individuals suffer a spinal cord injury (SCI) worldwide [1]. The consequence of an SCI is the partial or complete loss of motor, sensory, and vegetative functions [2]. As a result of an SCI, individuals may experience a loss of independence in mobility affecting their community participation and integration, leading to a decreased quality of life [3]. Balance may also be compromised after an SCI [4], which also affects walking ability [5,6]. Achieving walking ability that is functional, safe, and effective is of high importance in individuals who have an SCI [5], particularly an incomplete SCI (iSCI) [7]. Most patients with iSCIs may recover, to a certain extent, their neurological deficit [8], and approximately 41% of patients with the ability to stand or walk achieve unrestricted ambulatory function six months post-SCI [9].

Walking function recovery is tackled through several pharmacological, physical, robotic, and neurophysiological approaches in which precise evaluation of walking function is mandatory [10]. It is therefore desirable that valid, reliable, responsive [11], clinically useful [12], and internationally accepted [13] measurement tools are applied to assess changes in walking ability in people with SCIs.

In this regard, several articles have reviewed and classified walking ability outcome measures. In 2008, a systematic review published by Lam et al. [14] analyzed the psychometric properties of several widely used outcomes measures to assess walking ability in people with an SCI: the 10 meter walk test (10MWT), the six-minute walk test (6MWT), the timed up and go test (TUGT), the spinal cord injury functional ambulation inventory (SCI-FAI), the walking index for spinal cord injury (WISCI) II, the mobility (indoors and outdoors items) sub-scale of the spinal cord Independence measure III (SCIM III_IOMob_), and the locomotor-related items of the functional independence measure (FIM_L_). Similarly, with the aim of identifying the most clinically relevant outcome measures of general motor function after traumatic SCI, Labruyère et al. [13] conducted a systematic review in which specific ambulatory function outcome measures were included, comprising the WISCI II and several distance-related walking tests such as the eight-meter walk test, the 10MWT, and the 15 m walking speed or 50 foot walking test (50FWT). Nevertheless, the authors did not analyze the psychometric properties of the ambulatory outcome measures presented. Another systematic review published by Furlan et al. [15] focused on metrics to assess disability after traumatic SCI, also analyzing their psychometric properties, including some metrics related to walking function, such as the WISCI, TUGT, 6MWT, and 10MWT, but also others that assessed disability and included some items related to walking function such as the modified Barthel index, FIM, and SCIM.

Along with the systematic reviews published, walking ability outcome measures have also been reviewed from a consensus perspective. The European Multicenter Study of Human Spinal Cord Injury (EM-SCI) reviewed the status and psychometric properties of the WISCI II, 6MWT, 10MWT, and TUGT [9]. In addition, the National Institute on Disability and Rehabilitation Research (NIDRR) SCI Measures Meeting published a consensus of valid outcome measures for gait analysis in people with SCI [12], which reviewed the strengths and weaknesses of the WISCI II, 10MWT, 6MWT, FIM_L_, and the five times sit-to-stand test (FTSST), equally considering their construction, administration, population applicability, and psychometric properties.

Even though several authors have already reviewed the different outcome measures used to assess disability, general motor function and walking ability in population with SCI, there has been no update in the exclusive field of walking ability outcomes measures in SCI since the systematic review published by Lam et al. in 2008 [14]. Therefore, the aim of this paper was twofold: (1) to provide an updated qualitative review of walking ability outcome measures in SCI, regardless of the etiology of the injury, considering the diverse factors involving walking function, their generalization related to other measures, and their limitations; (2) to analyze their psychometric properties with the aim of providing reliable evidence to clinicians in the assessment of walking ability in SCI.

## 2. Materials and Methods

### 2.1. Search Strategy

A systematic review was conducted following the Preferred Reporting Items for Systematic Reviews and Meta-Analysis (PRISMA) 2009 statement, flow diagram and checklist. An electronic search of the literature was performed by two reviewers (IS-A. and AJd-A) using the databases PubMed, Cochrane, and PEDro, with no restriction on date of publication until 1 April 2020. Combinations of keywords (Medical Subject Headings—MeSH—and free terms), including truncation for the different variations of words, were connected by Boolean operators as follows: [(“spinal cord injury”) OR (“spinal cord” AND (“injured” OR “injuries”))] AND (“walking” OR “gait”) AND (“metric” OR “metrics” OR “scale” OR “scales” OR “test” OR “index” OR “score” OR “scores”); following an advanced search in the field “Title, Abstract” or “Title, Abstract, Keyword”.

### 2.2. Study Selection

The included articles fulfilled the following inclusion criteria: (i) studies including walking ability outcome measures in SCIs, also including balance; (ii) obtained by instrumental, numerical, or observational procedures; (iii) the latest version if there was more than one; (iv) analysis of at least one psychometric property of the measure; (v) no limitation on American Spinal Injury Association (ASIA) Impairment Scale (AIS) or Neurological Level of Injury (NLI) [2]; (vi) adult population with no age limit; (vii) written in English.

This systematic review excluded articles according to the following exclusion criteria: (i) studies published as conference proceedings and clinical trials registration; (ii) studies that used walking ability outcome measures to evaluate the results of an intervention but did not analyze their psychometric properties; (iii) systematic or no systematic reviews.

### 2.3. Data Collection

General characteristics of the studies, including sample size, injury features (SCI etiology, traumatic or non-traumatic, and type of AIS), population characteristics (age and gender), inclusion and exclusion criteria followed in the study, walking ability outcome measures studied, and statistical results of psychometric properties analyzed (validity, reliability, and responsiveness), were extracted. The evaluation criteria for defining the measurement properties and their standard values are shown in Table 1.

The screening of titles and abstracts obtained from the electronic search was followed by one reviewer (IS-A), who decided which articles met the inclusion criteria. For those included, the full-text articles were obtained, and two reviewers (IS-A and AJd-A) executed a new screening to confirm their relevance and to remove those that were not of interest to the review. Any disagreement on the selection of the articles was resolved by discussion with a third author (RC-d-l-C).

## 3. Results

**Figure 1:**
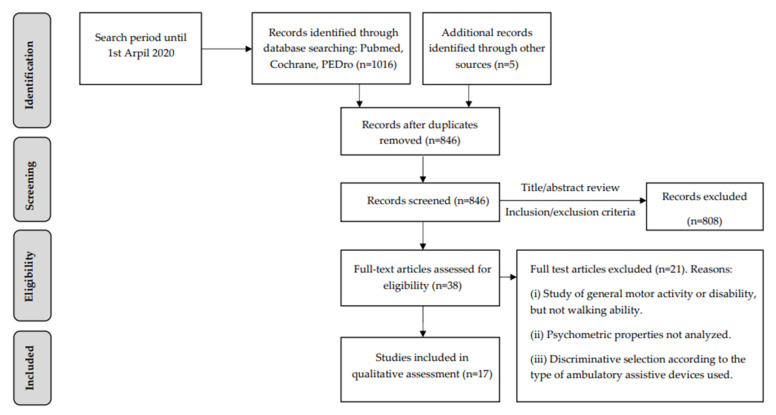
Flow diagram of the search strategy results.

The initial search of the databases yielded 1016 results, and five additional records were identified through other sources (website searches and citation tracking). After duplicate removal, 846 articles were screened applying the inclusion and exclusion criteria by reading the titles and abstracts. Eight hundred and eight articles were excluded and 38 were full-text screened for eligibility. Twenty-one articles were excluded (Figure 1) because of the following reasons: (i) not directly related to walking ability but to general motor activity or disability; (ii) psychometric properties were not analyzed; (iii) discriminative selection of walking ability outcome measures according to the type of ambulatory assistive devices (AADs). Finally, 17 articles were included in this review [4,10,11,16,18,19,20,21,22,23,24,25,26,27,28,29,30].

A total of 13 walking ability outcome measures were obtained and grouped into different categories (Table 2): the gait profile score (GPS), the 10MWT, the spinal cord injury functional ambulation profile (SCI-FAP), the TUGT, FTSST, 6MWT, WISCI, SCI-FAI, SCIM_IOMob_, the locomotor stages in spinal cord injury (LOSSCI), the Berg balance scale (BBS), the community balance and mobility (CB&M) scale, and the activity-based balance level evaluation (ABLE) scale. The search yielded three more outcome measures that were excluded due the lack of psychometric data, the Gillette gait index (GGI) and the 50FWT, or because its mobility sub-scale broadly measured ambulation and wheelchair propulsion, i.e., the FIM_L-_.

Five out the 17 articles analyzed the WISCI, four the 10MWT, and two the 6MWT, TUGT, and the BBS. Each one of the remaining walking ability outcome measures were analyzed in only one article (Table 3). The WISCI is the walking ability outcome measure which has psychometric properties that are the most assessed, while the GPS is the least. Table 4 shows the score for the psychometric properties.

### 3.1. Synthesis of the Results

#### 3.1.1. The Gait Profile Score

Three-dimensional gait analysis (3DGA) is considered the gold standard to evaluate walking abnormality and to assess changes after rehabilitation interventions [31].

Accordingly, the GPS is a single index outcome measure derived from 3DGA data that summarizes the overall quality of a patient’s kinematics by quantifying its deviation from a reference population without gait pathology [32]. The GPS is calculated from nine kinematic variables obtained from 3DGA (pelvic tilt, hip flexion, knee flexion, ankle dorsiflexion, pelvic obliquity, hip abduction, pelvic rotation, hip rotation, and foot progression) to provide the gait variable scores (GVSs), which root mean square average of all individual GVSs for a particular side equals the GPS, which is presented as left, right, and total.

#### 3.1.2. The 10 Meter Walk Test

The 10MWT measures the time invested in walking 10 m at a preferred or maximum walking speed [9] with physical assistance, orthoses, or any AAD required [12], to calculate walking speed. The 10MWT may be conducted in both static and dynamic start conditions: in the dynamic condition, two meters before starting the measure and two meters once reaching the end of the 10 m pathway, allow the individual to accelerate and decelerate [10].

#### 3.1.3. The Spinal Cord Injury Functional Ambulation Profile

The SCI-FAP [18] encompasses the timed performance of seven walking tasks at a comfortable walking pace: the TUGT, walking while carrying a bag, negotiating a carpet, obstacles, stairs, a step, and a door; a multiplication factor to quantify the AAD or physical assistance is needed for each task. The assistance rating may vary from one (independent person) to six (individual unable to complete the task).

#### 3.1.4. The Timed up and Go Test

The TUGT [33] measures the time needed to stand up from a chair, walking three meters, turning around a cone, and sit back on the chair, all at a maximum and safe speed, with or without AADs [9]. It is strongly correlated with balance, postural control, walking ability, and the risk of falls [33].

#### 3.1.5. The Five Times Sit-to-Stand Test

The FTSST records the time needed to, from sitting, stand up and sit back five times at the safe fastest speed without using external supports [34]. It has been applied to measure lower limb strength and balance control in several populations [34,35,36].

#### 3.1.6. The Six-Minute Walk Test

The 6MWT measures the distance recorded during six minutes walking at a preferred or maximum walking speed [9]. It was originally intended as an adaptation of the 12 min walk test (12MWT) to measure endurance in individuals with respiratory disease [37]. High correlation coefficients were found between the two-minute walk test (2MWT), 6MWT, and 12MWT, showing that they were similar measures of endurance [38].

#### 3.1.7. The Walking Index for Spinal Cord Injury (WISCI)

The WISCI is a walking scale specifically developed for the iSCI population. It was originally composed of 19 levels [39], but a latter revision in 2001 [40] modified the WISCI adding two more levels (WISCI II), integrating a hierarchical order for the use of AADs, orthoses, and the physical assistance needed to complete a 10 m walking distance. WISCI scores differs from self-selected (SS) WISCI, defined as the level the individual reports using to walk in the community or the household, and maximum WISCI, which is related to the highest level at which a person can safely walk 10 m [41].

#### 3.1.8. The Spinal Cord Injury Functional Ambulation Inventory

The SCI-FAI is an observational gait assessment instrument aimed at measuring functional walking ability in individuals with SCI through three categories of performance [27]: (i) gait parameters, (ii) use of AADs and orthoses (both ranked for each limb), and (iii) temporal–distance measures assessed by both the walking mobility scale (modified from a scale published by Perry et al. [42] and directed at assessing self-reported level of walking using a 0–5 score) and the 2MWT [38], which is included as a measure of walking speed and endurance. Since compound scores for each domain are intended to measure different fields of function, where higher scores indicate higher levels of function, they are not combined into an overall score.

#### 3.1.9. The Spinal Cord Independence Measure mobility items

The SCIM is a global disability scale developed by Catz et al. [43] for individuals with SCI in order to capture independence on performing ADL (activities of daily living), categorized into three areas of function: self-care, respiration and sphincter management, and mobility. It was revised by Catz et al. [44] in a new version (SCIM II), the latter resulting in the SCIM III [45]. SCIM_IOMob_, which assess walking ability, is classified in three main assessment groups: mobility indoors, mobility for moderate distances (0–100 m), and mobility outdoors (>100 m). All of them are similarly scored, from the requirement of total assistance (scored 0) to the independence of AADs, orthoses and personal supervision (scored 8).

#### 3.1.10. The Locomotor Stages in Spinal Cord Injury

The LOSSCI is a five-stage scale result of applying and adapting to SCI [28] the original Vojta’s 10 specific locomotor stages for children with cerebral palsy [46]. Each LOSSCI stage should be evaluated in ascending order and the grading is determined by the highest stage the person can accomplish: (i) orienting to and touching or grasping an object in supine position, (ii) trunk uprighting in prone position, (iii) creeping, (iv) crawling or walking with AAD, (v) independent bipedal locomotion. The person’s highest stage is reached when at least one item in a stage is achieved. The LOSSCI is based on the idea that the progress of people with SCI during rehabilitation is to some extent comparable to the typical development of ontogenic locomotion in children [28].

#### 3.1.11. The Berg Balance Scale

The BBS is a 14 item scale, originally designed to assess balance and fall risk in elderly population [47], that comprises sitting and standing balance tasks, but also transfers, reaching and turning tasks. Depending on the performance, each task is rated from 0 (unable to perform the task) to 4 points (best performance), with a total score ranging from 0 to 56 points.

#### 3.1.12. The Community Balance and Mobility Scale

The CB&M scale, originally developed and validated for the brain-injured population [48], is a 13 item scale (19 items for the total of right and left sides) that measures performance of balance and mobility tasks, some of them timed, which reflect motor skills needed for community participation. Each item is scored on a five-point ordinal scale (one item on a six-point ordinal scale), with higher scores indicating better performance [48].

#### 3.1.13. The Activity-based Balance Level Evaluation Scale

The ABLE scale, developed and tested to assess balance activities in population with SCI [30], was initially composed by 30 items and then refined to a total of 28 items across three functional domains: sitting, standing, and walking (which constitute seven items of the total). Since each item has distinct definitions, scores are not equal.

## 4. Discussion

There are several outcome measures used in clinical settings to assess walking ability and/or evaluate the effects of walking rehabilitation interventions in people with SCI. In the last decade, several authors conducted systematic reviews in the field of outcome measures used to assess disability [15], general motor function [13], and walking ability [14] in populations with SCIs. In general, psychometric properties were studied [14,15], but not in [13]. There has been no update since 2008 in the exclusive field of walking ability outcomes in SCI in which psychometric properties were also studied [14]. Moreover, despite all the already published systematic reviews [13,14,15] and joint efforts [9,12], there is no clear consensus on which measure, or combination of measures, can yield a comprehensive and clinically relevant information on walking function.

To be easily and broadly applied in clinics, the assessment procedures must be timely, affordable, and with no need of sophisticated, thus complicated, equipment. The evaluator has to choose the metric, or set of metrics, within all the broad range of possibilities for measuring changes in walking ability, including balance, which may be challenging due to the number, heterogeneity, and differences in construct of the available metrics. Psychometric properties of each outcome measure, assessment time, cost of the specialized equipment, and human resources required, not only to accomplish the test but also to analyze the results, are relevant aspects that may determine the choice. Furthermore, some metrics seem to be redundant. In this sense, this paper aimed to bring a qualitative review of the different walking outcome measures which are currently available to measure balance and ambulation in people with an SCI to provide a useful and reliable guide in clinical and research settings.

With regard to the outcome measures included in this review, 3DGA provides specific information to guide rehabilitation interventions to improve walking function of people with traumatic and non-traumatic iSCIs, quantifying changes in gait kinematics and, hence, the impact on the kinematics of the intervention [31]. Concerning the GPS and GVSs, although they have shown an excellent inter- and intra-session reliability [22], they have some limitations, since they do not comprise movement timing nor the gait deviation direction. In addition, as individual gait scores, they are not directly comparable because they may not have the same clinical meaning [22], e.g. ankle kinematics has less clinical importance than foot progression on overall walking function in SCI. However, 3DGA is both time and cost expensive, and the large quantity of information provided is sometimes useless when related to the purpose of the assessment. Therefore, categorical- and spatiotemporal-related walking measures can be considered to assess walking ability.

The 10MWT and 6MWT allow to assess functional ambulation in which gait speed over short distances (10MWT) is thought to represent crossing the street, while longer distances (6MWT) reflect endurance required for community ambulation [49]. Both tests have shown to be reliable [10,19,23]—the 10MWT in both static and dynamic start conditions [10], valid [19,23], and responsive [11] outcome measures to assess walking ability in people with SCI. Nevertheless, sensitivity of the 6MWT and 10MWT may be affected by a floor effect among patients who cannot walk for six minutes or ambulate 10 m, and a ceiling effect in patients who can continue walking beyond six minutes at the same pace or walking much farther than 10 m with the same walking speed [9,12]. The 10MWT is both time and cost effective and requires no special equipment to administer. The 6MWT involves, nevertheless, greater time investment and a specific environment where the pathway should contain as few turns as possible [9], since shorter track lengths may decrease the distance walked. Likewise, it is described that different levels of verbal encouragement may make significant differences in the 6MWT performance [50] and that repeated testing might produce a training effect and improve individuals’ performance [23,37], because people become rapidly familiarized with the 6MWT [9]. However, in either case, information of basic yet important components of walking production is missing, such as joint kinematics and/or limb coordination.

Concerning the TUGT, since it encompasses a more complex timed set of tasks, such as standing up, walking, turning and sitting down, it might better reflect community walking ability [9]. The TUGT has demonstrated to be a valid and reliable outcome measure as well to assess walking ability in individuals with SCI [19,23]. No ceiling effect has been found for the TUGT, yet a floor effect exists in cases in which the person is not able to stand and sit on the chair independently. Although the TUGT is a quick test and it does not require special equipment or training, as it combines several tasks in one test, it might decrease the sensitivity of the information gained [9]. Similar to the 6MWT, it is recommended to perform a test trial at least once before conducting the TUGT [9], since individuals become rapidly familiarized with the test [23]. Regarding the FTSST, which has also shown to be a valid and reliable outcome measure to assess levels of independence in ambulatory individuals with SCI [19], it is more demanding in terms of lower limb strength and balance control than the TUGT and, therefore, may also have a floor effect in individuals who have not recovered yet the ability to perform the task required independently [19].

While the previous spatiotemporal-related walking and balance measures can be applied in a simplified yet standardized environment, the SCI-FAP evaluates walking performance on a variety of common walking skills which are representative of real-life scenarios [51]. Furthermore, each task of the SCI-FAP can be assessed independently because each one has shown to be a valid and reliable tool [18]. However, the SCI-FAP shows a ceiling effect, since it does not discriminate between individuals who walk at normal speeds without AADs from those with physical assistance. Conversely, the floor effect is minimized by setting high maximum times for the tasks [18]. Because the SCI-FAP neither distinguishes among the different levels of physical assistance nor considers the use of orthoses, and individuals are required to be able to walk at least five meters, future work with a larger sample should focus on confirming the responsiveness of the SCI-FAP in individuals with a lower level walking ability [51].

The WISCI is a widely used scale in SCIs in which levels are ordered by degree of the underlying person’s impairment, from most impaired to least impaired [26], considering the person’s needs in terms of physical assistance, AADs, and orthoses to walk 10 m. Nevertheless, ranking categories have been questioned, suggesting the classification in terms of levels of independent walking rather than the requirement of external assistance [23]. Although the WISCI is a simple and time-effective outcome measure that does not require any equipment, some authors have criticized the fact that WISCI does not incorporate elements of speed or endurance [11,25,39] and, thus, it has been suggested to combine it with the 10MWT due to the fact of its quicker implementation [9,11]. Likewise, the WISCI does not provide information concerning joint kinematics relating to limb coordination or spatiotemporal parameters. Nevertheless, the WISCI II is a valid [25,26], reliable—in both SS and maximum WISCI II levels [16,24]—and responsive [11] outcome measure to assess walking ability in people with SCI. It has shown a ceiling effect [20,29] and a better sensitivity to change in persons with more impaired gait compared to those with higher levels of walking function in which the 6MWT and 10MWT are more sensitive to measure changes [11].

Regarding the SCIM, since it is a disability scale for people with SCI, it is not a specific scale for ambulation. Nevertheless, the SCIM II_IOMob_ has shown to be a valid and responsive outcome measure to assess the efficacy of new interventions on ambulatory function in people with SCI [21]. Furthermore, SCIM_IOMob_ ranks in terms of levels of independent walking in which independency from AADs is ranked higher in comparison with the WISCI [26]. Future works should test validity, reliability, and responsiveness of indoors and outdoors items of the latest version (SCIM III) as a walking outcome measure in SCI.

The SCI-FAI is a valid, reliable, and sensitive outcome measure of walking ability for individuals with SCI [27] that attempts to combine the use of AADs and orthoses, such as the WISCI does, with the use of gait scores to assess spatiotemporal parameters, a distance measure (2MWT), and an independence walking scale. Nevertheless, since it is an observational gait assessment instrument, gait parameters section may take longer to assess and have a subjective bias, which does not occur with 3DGA. Furthermore, with the exception of the 2MWT, the SCI-FAI has also shown a ceiling effect [29].

As alternative tool to all the walking ability outcome measures above mentioned, the LOSSCI allows to assess the locomotor progress of a person with SCI from an ontogenic point of view, and it has shown to be a valid and reliable outcome measure [28]. Concerning bipedal stance, LOSSCI conceives the use of AADs and includes items of independent locomotion such as walking on flat surfaces and stopping on command, walking up an incline, and one-legged standing. Nevertheless, although it is time-effective and does not require special equipment, it has some limitations such as that the use of orthoses is not conceived and that it does not measure time invested in walking nor distance covered. Furthermore, a ceiling effect may appear in people with higher levels of walking function as well.

Finally, balance assessment has been classically ignored as an essential component of ambulation in people with SCI. Besides the TUGT and the FTSST, which involve a balance component, there is not a wide range of balance outcome measures in the literature evidencing their validity in SCI. The most popular is the BBS, which has shown to be a valid [20,29] and reliable [20] outcome measure to assess balance in people with SCI. Nevertheless, the BBS score is not associated with the number of falls of people with SCI and it is not able to discriminate fallers from non-fallers [20]. Likewise, it has shown a ceiling effect [20,29]. In this respect, the CB&M scale has not shown a ceiling effect and is a valid outcome measure when assessing walking balance in people with iSCI and mild walking impairment [4]. It incorporates more complex mobility tasks that require precision and accuracy, better reflecting real-life situations. The CB&M scale has shown an excellent internal consistency but not sufficiently high to suggest redundancy among the test items [4]. Lastly, as an alternative to the previous balance measures, the ABLE scale was created as a specific outcome measure of balance in SCI [30]. The diversity of items and difficulties allows assessing the person throughout the recovery process, from sitting to walking. Nonetheless, despite the minimal floor and ceiling effects, the walking tasks performed are not as challenging as those encountered in the CB&M scale [4]. Further studies are required to reduce the 28 items to avoid overlap in levels of difficulty and to decrease the time invested to administer the scale, to examine its reliability, and to determine the sensitivity or specificity needed to predict fallers in people with SCI [30].

Walking ability is a complex set of functional tasks involving static and dynamic balance, joint kinematics, limb coordination, changes of speed, and endurance requirements to allow the person adapting to a changeable environment present in real-life situations. From this standpoint, it does not make sense to divide walking assessment in different outcome measures but evaluating walking function with a single outcome measure able to capture walking complexity as a whole. Nevertheless, the findings of this review showed several outcome measures which assess different relevant aspects involved in walking ability from an isolated point of view. Therefore, the authors suggest combining several metrics, without redundancy, to provide a global assessment of walking function.

Since 3DGA is the gold standard to measure improvements in walking function quantifying changes in gait kinematics [31], kinematic data might be complemented by the inclusion of spatiotemporal-related walking and balance measures, as well as categorical measures of ambulation. Nevertheless, due to the lack of validity of multivariate walking metrics built upon 3DGA data, such as the GPS and GVSs, further work is required to validate them. Future research should focus in assessing correlations with the 10MWT, TUGT, and WISCI II, since they are valid and reliable walking outcome measures in SCI but also easy to perform, time- and cost-effective, and they measure relevant aspects such as the time invested in walking, balance, and AADs, orthoses, and physical assistance required in walking ability. We consider that these measures might be a good combination of metrics to cover the whole functional spectrum of walking ability. However, aware that 3DGA is both time-consuming and costly, we also consider dismissing kinematic data in cases where this information can be useless related to the purpose of the assessment, and using the remaining combination of outcome measures proposed. Nevertheless, none of these metrics include environmental factors related to real-life situations. In this sense, the SCI-FAP [18] might be a good option to complement the set of outcome measures proposed. To avoid redundancies, future work should consider integrating SCI-FAP tasks within the 3DGA protocol, providing a more natural walking task, and also implementing other motion capture technologies such as wearable inertial sensors as an alternate to 3DGA with digital photogrammetry.

In any case, a selection and a combination of outcome measures to assess walking and balance in people with SCI should be a choice of the clinician according to the purpose of the assessment, the level of walking function of the person evaluated in pursuit of avoiding ceiling or floor effects of the metric, time invested in performing the test, and technical and human resources required.

The main strength of this work is the contribution with an updated review of all the outcome measures available in the published literature to assess walking ability in SCI, considering the diverse factors involving walking function and their limitations, and analyzing their psychometric properties. Nevertheless, this systematic review presents some methodological limitations: (i) articles included were not classified according to the levels of evidence and grades of recommendation for diagnosis studies established by the Oxford Center for Evidence-Based Medicine [52]; (ii) meta-analysis of the data was not conducted; (iii) only the latest versions of outcome measures were considered; (iv) some outcome data may have been missed during collection; (v) the results of this work may have been influenced by language restrictions. The levels of evidence of the articles included and meta-analysis of the data were not considered related to the purpose of the study. Therefore, our conclusions should be interpreted with caution.

## 5. Conclusions

This paper provides an updated review of the walking ability outcome measures that are currently available to measure ambulation in people with SCI, their psychometric properties, and their limitations. The choice of a single or a set of outcome measures will be determined by the clinician depending on the purpose of the assessment and the financial, human, and time resources required. New outcome measures of walking ability (the GPS, SCI-FAP, FTSST, LOSSCI, BBS, and CB&M and ABLE scales), not envisaged in previous systematic reviews in the published literature, should be considered to assess walking ability in individuals with SCI, considering thus different functional perspectives related to more realistic daily life situations. Since walking ability is a complex set of functional tasks, the authors propose a combination of 3DGA with a minimum of valid and reliable outcome measures, such as the 10MWT, TUGT, and WISCI, to cover the whole functional spectrum of walking ability. Future work is required to validate multivariate walking metrics such as the GPS, and to integrate SCI-FAP tasks in the 3DGA protocol, providing a more realistic environment for walking assessment.

## Figures and Tables

**Table 1:** Evaluation criteria and standards.

Property	Definition	Standard Value
Reliability	Reproducibility: degree to which the score is free from random error (including test re-test reliability, intra- and interrater reliability) [14]. Internal consistency: homogeneity of the items [14].	Intra- and interrater reliability (Spearman or Pearson coefficients, k coefficient ^1^, ICC ^2^): ≥0.75 excellent, 0.40–0.74 moderate, ≤0.39 poor [14]. Test re-test reliability: SRD ^3^ [16]. Cronbach’s α: ≥0.80 excellent, 0.70–0.79 adequate, ≤0.69 poor [14].
Validity	Assessing if the instrument actually measures what it intends to measure [14]. Criterion validity (concurrent, convergent, predictive) is the extent to which scores on a particular questionnaire relate to a gold standard. For most of the functional scales, there was no criterion standard and, hence, construct validity was used [17].	Jaspen coefficient of multiserial correlation (M) [18], point biserial correlation coefficient (rpb) [19], Spearman (ρ) or Pearson (r) coefficients: ≥0.70 excellent, 0.50–0.69 moderate, ≤0.49 poor [14].
Responsiveness	The ability of a questionnaire to detect clinically important changes over time [17]. Floor or ceiling effect: the number of respondents who achieved the lowest (floor) or highest (ceiling) possible score [17].	SRD ^3^ [14], ROC analysis ^4^ [20], *p*^−^value ^5^ [11], SRM ^6^ (0.20 small, 0.50 medium, >0.80 large responsiveness), linear regression analysis [21]. Problematic when >20% of subjects received either minimum or maximum scores [14].

^1^ Cohen’s kappa coefficient (k), ^2^ intraclass correlation coefficient (ICC), ^3^ smallest real difference (SRD), ^4^ area under curve (ROC analysis), ^5^ statistical significance (*p*-value), and ^6^ standardized response mean (SRM).

**Table 2:** Categories of walking ability outcome measures in SCIs.

Categories	Outcome Measures
**Multivariate walking metrics**	GPS ^1^
**Spatiotemporal-related walking/balance measures**	
Timed measures	
Speed-related walking tests	10MWT ^2^
	SCI-FAP ^3^
Speed-related balance tests	TUGT ^4^
	FTSST ^5^
Distance measures (endurance-related walking tests)	6MWT ^6^
**Categorical measures of ambulation**	
Walking assessment measures	WISCI ^7^
	SCI-FAI ^8^
Multidimensional measures (locomotor-related subscales)	SCIM_IOMob_^9^
	LOSSCI ^10^
Balance measures	BBS ^11^
	CB&M ^12^ scale
	ABLE ^13^ scale

^1^ Gait profile score (GPS), ^2^ 10 meter walk test (10MWT), ^3^ spinal cord injury functional ambulation profile (SCI-FAP), ^4^ timed up and go test (TUGT), ^5^ five times sit-to-stand test (FTSST), ^6^ six-minute walk test (6MWT), ^7^ walking index for spinal cord injury (WISCI), ^8^ spinal cord injury functional ambulation inventory (SCI-FAI), ^9^ spinal cord independence measure indoors/outdoors mobility items (SCIM_IOMob_), ^10^ locomotor stages in spinal cord injury (LOSSCI), ^11^ Berg balance scale (BBS), ^12^ community balance and mobility (CB&M), ^13^ activity-based balance level evaluation (ABLE).

**Table 3:** Main results of the systematic review.

Reference	Outcome Measures	Sample Size	Injury Features: Etiology (E) AIS ^1^	Population: Age (Mean ± SD) Gender (M/F) ^2^	Inclusion/Exclusion Criteria (IC/EC)	Psychometric Properties	Results
Wedege et al. [22]	GPS	15	E: Traumatic and non-traumatic. AIS ^1^: D.	Age range: 25–62 11 M/4 F.	IC: ≥ 1 year post-injury, ability to walk 10 m without personal assistance. EC: other diseases affecting gait; BTIs ^3^ and orthopedic treatment or neurosurgery in the lower limbs within the last 3–6 months.	Inter-and intra-session reliability.	ICC ≥ 0.93 (intersession, except hip rotation), ≥0.96 (intrasession).
Van Hedel et al. [23]	10MWT 6MWT TUGT	22 75	E: Traumatic and ischemic. AIS ^1^: A-D.	Reliability group: 52 ± 20 14 M/8 F. Validity group: 54 ± 20 45 M/30 F.	IC: WISCI II > 0 and no additional gait impairments.	Inter- and intrarater reliability and concurrent validity.	r > 0.97; |r| > 0.88 (10MWT, 6MWT, and TUGT correlated between each other), |ρ| > 0.60 (correlated with WISCI II).
Poncumhak et al. [19]	10MWT TUGT FTSST	66 16	E: Traumatic and non-traumatic. AIS ^1^: C, D.	Validity groups: FIM_L_^4^ 6: 50.9 ± 13.4 22 M/11 F. FIM_L_^4^ 7: 50.2 ± 9.5 24 M/9 F. Reliability group: 50.8 ± 10.3 11 M/5 F.	IC: ability to stand up independently and to walk at least 50 m with or without AADs ^5^ (FIM_L_^4^ scores 6–7).	Concurrent validity and interrater reliability.	rpb = 0.78, −0.69, −0.60 (10MWT, TUGT, and FTSST correlated with FIM_L_^4^, respectively); ICC = 0.997–1.00
Van Hedel et al. [11]	WISCI II 6MWT 10MWT	22	E: Traumatic and non-traumatic. AIS ^1^: N/A ^6^.	45.5 ± 16.7 18 M/4 F.	IC: functional ambulation within the first month after injury (WISCI II ≥ 1).	Responsiveness.	WISCI II (over the first 3 months): *p* = 0.005; 6MWT and 10MWT (over the first 6 months): *p* < 0.001–0.01
Scivoletto et al. [10]	10MWT	37	E: Traumatic and non-traumatic. AIS ^1^: C, D.	Age range: 19–77 28 M/9 F.	IC: functional ambulation at home or community, with or without the use of AADs ^5^. EC: cognitive deficit, cardiac or lung diseases.	Inter- and intrarater reliability.	ICC = 0.95–0.99; *p* = 0.09 (in both dynamic and static start conditions).
Musselman et al. [18]	SCI-FAP	32 60 able-bodied.	E: N/A ^6^. AIS ^1^: C, D.	47.6 ± 14.2 24 M/8 F. 42.9 ± 16.0 34 M/26 F.	IC: ≥ 6 months after injury, ability to walk ≥ 5 m with or without physical assistance and/or AADs ^5^, free of any disease and changes in medications affecting walking ability, not receiving walking training. IC: > 18 years, ≤ 1 fall in the previous month, free of any disease affecting walking ability.	Interrater and test-retest reliability, internal consistency, convergent and discriminative validity.	ICC = 1.00 (interrater), 0.98 (test-retest); α = 0.95; r = −0.59 (correlated with 10MWT and 6MWT), M = 0.68 (correlated with WISCI II), higher scores in injured individuals related to able-bodied ones.
Marino et al. [24]	WISCI II	26	E: Traumatic and non-traumatic. AIS ^1^: A, C, and D.	46.4 ± 19.3 16 M/10 F.	IC: ≥ 6 months after injury, independent lower limb weight bearing once a week. EC: SS ^7^ WISCI < 6 or equal to 20, any other medical condition which could limit safety ambulation.	Intra- and interrater reliability.	SS ^7^ WISCI: ICC = 1.00 (intra- and interrater). Maximum WISCI: ICC = 1.00 (intra-), 0.98 (interrater).
Morganti et al. [25]	WISCI II	76	E: Traumatic and non-traumatic. AIS ^1^: A-D.	50.4 ± 19.3 184 M/100 F.	IC: WISCI > 0 and < 20.EC: cognitive impairments that disable to participate in the rehabilitation program.	Concurrent validity.	Correlation with SCIM IMob items (ρ = 0.97) and FIM_L_^4^ (ρ =0.70).
Ditunno et al. [26]	WISCI II	146	E: Traumatic. AIS ^1^: B-D.	Age range: 16–69 78% M/22% F.	IC: subjects within 8 weeks of onset of injury and within 1 week of admission for rehabilitation. EC: FIM_L_^4^ > 3.	Concurrent and predictive validity.	Correlations at 6 months with BBS (ρ = 0.90), FIM_L_^4^ (ρ = 0.89), and 6MWT (ρ = 0.79).
Scivoletto et al. [16]	WISCI II	33	E: Traumatic. AIS ^1^: C, D.	Median age: 44. 28 M/5 F.	IC: subjects within 3 months of onset of injury, with a motor level of C4-L1 inclusive.	Intra- and interrater reliability, and test re-test reliability.	Maximum WISCI II scores: ICC = 0.975–0.999; SRD = 1.15, 1.68 (tetra- and paraplegics, respectively).
Field-Fote et al. [27]	SCI-FAI	22 19	E: N/A ^6^. AIS ^1^: N/A ^6^.	Validity/reliability group: 32 ± 13 17 M/5 F. Sensitivity group: 31.7 ± 9.4 13 M/6 F.	IC: ability to maintain stance on the weight-bearing limb independently and to take at least 8 steps using any AADs ^5^. IC: N/A ^6^.	Intra- and interrater reliability, convergent validity, and sensitivity.	ICC = 0.70–0.96; r = −0.74, −0.70 (gait score correlated with the 10 feet-walking speed); r = 0.58 (gait score correlated with LEMS ^8^ to assess sensitivity).
Van Hedel et al. [21]	SCIM II IOMob items	886	E: Traumatic. AIS ^1^ A: 413 AIS ^1^ B: 113 AIS ^1^ C: 137 AIS ^1^ D: 223	39 ± 18; 19% F. 42 ± 18; 27% F. 48 ± 20; 32% F. 47 ± 17; 22% F.	IC: patients classified with AIS at 1 month after injury and assessed at least at 2 successive time points (at 2 weeks and 1, 3, 6, and 12 months after injury) with SCIM II and either the 10MWT or WISCI II.	Concurrent, validity, internal, and external responsiveness.	IMob items correlated with 10MWT and WISCI II in AIS C-D: ρ = 0.75–0.91; SRM = 0.67–1.24 (IOMob items in AIS C-D); linear regression analysis = 0.79 (IMob items correlated with 10MWT in AIS C).
Maurer-Burkhard et al. [28]	LOSSCI	65 161	E: N/A ^6^. AIS ^1^: C, D. E: N/A ^6^. AIS ^1^: A-D.	Reliability group: 44.9 ± 16.0 77% M/23% F. Validity group: 48.3 ± 20.2 65.8% M/34.2% F.	IC: 18–80 years, ≥ 8 weeks after injury, having been assessed by 2 raters in 2 successive assessments within 1–5 days. IC: SCIM databases from the EM-SCI ^9^ obtained within the first year after injury.	Interrater reliability and construct validity.	WCk ^10^: 0.98; ρ = 0.77–0.82 (correlated with SCIM IOMob items).
Lemay et al. [29]	BBS	32	E: Traumatic and non-traumatic. AIS ^1^: D.	47.9 ± 12.8 25 M/7 F.	IC: ability to walk 10 m independently with or without AADs ^5^. EC: other neurological conditions or existence of walking difficulties before the injury.	Concurrent validity and responsiveness.	ρ = 0.71–0.82 (correlated with the SCI-FAI, WISCI II, 10MWT and TUGT); ceiling effect: 44.8% (WISCI II), 68.8% (gait score SCI-FAI), 34.4% (BBS and Walking Mobility and assistive devices section of SCI-FAI).
Wirz et al. [20]	BBS	42	E: Traumatic and non-traumatic. AIS ^1^: A-D.	49.3 ± 11.5 33 M/9 F.	IC: ≥ 1 year after injury prior to enrollment, ability to walk unless 15 m. EC: < 18 or > 65 years, vestibular or visual systems impairments, and others affecting standing or walking function.	Construct validity, interrater reliability, and responsiveness.	ρ = −0.82, −0.89, −0.93 (correlated with WISCI II, SCIM II Mob items, and 10MWT, respectively); ICC = 0.95; ROC = 0.48 (95% confidence interval = 0.29–0.67), ρ = −0.17 (number of falls), ceiling effect: ±1/3 of subjects (BBS, WISCI II, SCIM II).
Chan et al. [4]	CB&M scale	30	E: Traumatic and non-traumatic. AIS ^1^: C, D.	38.3 ± 15.3 23 M/7 F.	IC: < 65 years, FIM ^4^ ≥ 115 at discharge, ability to complete the CB&M scale and any other balance or walking outcome measures within one week of each other. EC: significant comorbid condition.	Convergent validity, internal consistency.	r = 0.47–0.72 (correlation with the 6MWT, 10MWT and BBS); α = 0.87
Ardolino et al. [30]	ABLE scale	104	E: Traumatic. AIS ^1^: A–D.	38.6 ± 15.0 79 M/25 F.	IC: ≥ 16 years, traumatic origin of the injury. EC: inability to tolerate upright supported sitting for at least 1 min, need for a spinal stabilization device, limited ability to bend or rotate, inability to follow 2-step commands.	Responsiveness.	Minimal floor and ceiling effects.

^1^ American Spinal Injury Association Impairment Scale (AIS), ^2^ male (M)/female (F), ^3^ botulinum toxin A injections (BTIs), ^4^ locomotor-related items of the functional independence measure (FIM_L_), ^5^ ambulatory assistive devices (AADs), ^6^ no available data (N/A), ^7^ self-selected (SS), ^8^ lower extremity motor score (LEMS), ^9^ European Multicenter Study of Human Spinal Cord Injury (EM-SCI), ^10^ weighted Cohen’s kappa coefficient (WCk).

**Table 4:** Psychometric properties of walking ability outcome measures in SCI.

	Reliability					Validity			Responsiveness
	Inter- and Intrasession	Test Re-Test	Intrarater	Interrater	Internal Consistency	Concurrent Convergent	Construct	Predictive	Floor/Ceiling Effects
GPS	+++								
10MWT			+++	+++		++/+++			*
SCI-FAP		+++		+++	+++	++			Ceiling
TUGT			+++	+++		++/+++			
FTSST				+++		++			
6MWT			+++	+++		++/+++			*
WISCI II		++	+++	+++		+++		+++	* Ceiling
SCI-FAI			+++	++/+++		+++			++ Ceiling
SCIM II_IOMob_						+++			++/+++
LOSSCI				+++			+++		
BBS				+++		+++	+++		+ Ceiling
CB&M scale					+++	+/++/+++			
ABLE scale									Ceiling/Floor

* Depending on the time after injury; +++ excellent/optimal/large; ++ moderate/adequate/good/medium; + poor.

## Data Availability

Not applicable.

## References

[1] Singh A., Tetreault L., Kalsi-Ryan S., Nouri A., Fehlings M.G. (2014). Global Prevalence and Incidence of Traumatic Spinal Cord Injury. Clin. Epidemiol..

[2] Kirshblum S.C., Burns S.P., Biering-Sorensen F., Donovan W., Graves D.E., Jha A., Johansen M., Jones L., Krassioukov A., Mulcahey M. (2011). International Standards for Neurological Classification of Spinal Cord Injury (Revised 2011). J. Spinal Cord Med..

[3] Anderson K.D. (2004). Targeting Recovery: Priorities of the Spinal Cord-Injured Population. J. Neurotrauma.

[4] Chan K., Guy K., Shah G., Golla J., Flett H.M., Williams J., Musselman K.E. (2017). Retrospective Assessment of the Validity and Use of the Community Balance and Mobility Scale among Individuals with Subacute Spinal Cord Injury. Spinal Cord.

[5] Barbeau H., Nadeau S., Garneau C. (2006). Physical Determinants, Emerging Concepts, and Training Approaches in Gait of Individuals with Spinal Cord Injury. J. Neurotrauma.

[6] Scivoletto G., Romanelli A., Mariotti A., Marinucci D., Tamburella F., Mammone A., Cosentino E., Sterzi S., Molinari M. (2008). Clinical Factors That Affect Walking Level and Performance in Chronic Spinal Cord Lesion Patients. Spine.

[7] Ditunno P.L., Patrick M., Stineman M., Ditunno J.F. (2008). Who Wants to Walk? Preferences for Recovery after SCI: A Longitudinal and Cross-Sectional Study. Spinal Cord.

[8] Spiess M.R., Müller R.M., Rupp R., Schuld C., van Hedel H.J.A., EM-SCI Study Group (2009). Conversion in ASIA Impairment Scale during the First Year after Traumatic Spinal Cord Injury. J. Neurotrauma.

[9] Van Hedel H.J.A., Wirz M., Dietz V. (2008). Standardized Assessment of Walking Capacity after Spinal Cord Injury: The European Network Approach. Neurol. Res..

[10] Scivoletto G., Tamburella F., Laurenza L., Foti C., Ditunno J.F., Molinari M. (2011). Validity and Reliability of the 10-m Walk Test and the 6-Min Walk Test in Spinal Cord Injury Patients. Spinal Cord.

[11] Van Hedel H.J.A., Wirz M., Curt A. (2006). Improving Walking Assessment in Subjects with an Incomplete Spinal Cord Injury: Responsiveness. Spinal Cord.

[12] Jackson A.B., Carnel C.T., Ditunno J.F., Read M.S., Boninger M.L., Schmeler M.R., Williams S.R., Donovan W.H. (2008). Gait and Ambulation Subcommittee Outcome Measures for Gait and Ambulation in the Spinal Cord Injury Population. J. Spinal Cord Med..

[13] Labruyère R., Agarwala A., Curt A. (2010). Rehabilitation in Spine and Spinal Cord Trauma. Spine.

[14] Lam T., Noonan V.K., Eng J.J., SCIRE Research Team (2008). A Systematic Review of Functional Ambulation Outcome Measures in Spinal Cord Injury. Spinal Cord.

[15] Furlan J.C., Noonan V., Singh A., Fehlings M.G. (2011). Assessment of Disability in Patients with Acute Traumatic Spinal Cord Injury: A Systematic Review of the Literature. J. Neurotrauma.

[16] Scivoletto G., Tamburella F., Laurenza L., Torre M., Molinari M., Ditunno J.F. (2014). Walking Index for Spinal Cord Injury Version II in Acute Spinal Cord Injury: Reliability and Reproducibility. Spinal Cord.

[17] Terwee C.B., Bot S.D.M., de Boer M.R., van der Windt D.A.W.M., Knol D.L., Dekker J., Bouter L.M., de Vet H.C.W. (2007). Quality Criteria Were Proposed for Measurement Properties of Health Status Questionnaires. J. Clin. Epidemiol..

[18] Musselman K., Brunton K., Lam T., Yang J. (2011). Spinal Cord Injury Functional Ambulation Profile: A New Measure of Walking Ability. Neurorehabil. Neural Repair.

[19] Poncumhak P., Saengsuwan J., Kamruecha W., Amatachaya S. (2013). Reliability and Validity of Three Functional Tests in Ambulatory Patients with Spinal Cord Injury. Spinal Cord.

[20] Wirz M., Müller R., Bastiaenen C. (2010). Falls in Persons with Spinal Cord Injury: Validity and Reliability of the Berg Balance Scale. Neurorehabil. Neural Repair.

[21] Van Hedel H.J.A., Dietz V., European Multicenter Study on Human Spinal Cord Injury (EM-SCI) Study Group (2009). Walking during Daily Life Can Be Validly and Responsively Assessed in Subjects with a Spinal Cord Injury. Neurorehabil. Neural Repair.

[22] Wedege P., Steffen K., Strøm V., Opheim A.I. (2017). Reliability of Three-Dimensional Kinematic Gait Data in Adults with Spinal Cord Injury. J. Rehabil. Assist. Technol. Eng..

[23] Van Hedel H.J., Wirz M., Dietz V. (2005). Assessing Walking Ability in Subjects with Spinal Cord Injury: Validity and Reliability of 3 Walking Tests. Arch. Phys. Med. Rehabil..

[24] Marino R.J., Scivoletto G., Patrick M., Tamburella F., Read M.S., Burns A.S., Hauck W., Ditunno J. (2010). Walking Index for Spinal Cord Injury Version 2 (WISCI-II) with Repeatability of the 10-m Walk Time: Inter- and Intrarater Reliabilities. Am. J. Phys. Med. Rehabil.

[25] Morganti B., Scivoletto G., Ditunno P., Ditunno J.F., Molinari M. (2005). Walking Index for Spinal Cord Injury (WISCI): Criterion Validation. Spinal Cord.

[26] Ditunno J.F., Barbeau H., Dobkin B.H., Elashoff R., Harkema S., Marino R.J., Hauck W.W., Apple D., Basso D.M., Behrman A. (2007). Validity of the Walking Scale for Spinal Cord Injury and Other Domains of Function in a Multicenter Clinical Trial. Neurorehabil. Neural Repair.

[27] Field-Fote E.C., Fluet G.G., Schafer S.D., Schneider E.M., Smith R., Downey P.A., Ruhl C.D. (2001). The Spinal Cord Injury Functional Ambulation Inventory (SCI-FAI). J. Rehabil. Med..

[28] Maurer-Burkhard B., Smoor I., von Reumont A., Deckstein G., Stierle I., Rupp R., Schuld C. (2016). Validity and Reliability of a Locomotor Stage-Based Functional Rating Scale in Spinal Cord Injury. Spinal Cord.

[29] Lemay J.-F., Nadeau S. (2010). Standing Balance Assessment in ASIA D Paraplegic and Tetraplegic Participants: Concurrent Validity of the Berg Balance Scale. Spinal Cord.

[30] Ardolino E.M., Hutchinson K.J., Pinto Zipp G., Clark M., Harkema S.J. (2012). The ABLE Scale: The Development and Psychometric Properties of an Outcome Measure for the Spinal Cord Injury Population. Phys. Ther..

[31] Murphy A.T., Kravtsov S., Sangeux M., Rawicki B., New P.W. (2019). Utilizing Three Dimensional Clinical Gait Analysis to Optimize Mobility Outcomes in Incomplete Spinal Cord Damage. Gait Posture.

[32] Baker R., McGinley J.L., Schwartz M.H., Beynon S., Rozumalski A., Graham H.K., Tirosh O. (2009). The Gait Profile Score and Movement Analysis Profile. Gait Posture.

[33] Podsiadlo D., Richardson S. (1991). The Timed “Up & Go”: A Test of Basic Functional Mobility for Frail Elderly Persons. J. Am. Geriatr. Soc..

[34] Whitney S.L., Wrisley D.M., Marchetti G.F., Gee M.A., Redfern M.S., Furman J.M. (2005). Clinical Measurement of Sit-to-Stand Performance in People with Balance Disorders: Validity of Data for the Five-Times-Sit-to-Stand Test. Phys. Ther..

[35] Meretta B.M., Whitney S.L., Marchetti G.F., Sparto P.J., Muirhead R.J. (2006). The Five Times Sit to Stand Test: Responsiveness to Change and Concurrent Validity in Adults Undergoing Vestibular Rehabilitation. J. Vestib. Res..

[36] Lord S.R., Murray S.M., Chapman K., Munro B., Tiedemann A. (2002). Sit-to-Stand Performance Depends on Sensation, Speed, Balance, and Psychological Status in Addition to Strength in Older People. J. Gerontol. A Biol. Sci. Med. Sci..

[37] Brooks D., Solway S., Gibbons W.J. (2003). ATS Statement on Six-Minute Walk Test. Am. J. Respir. Crit. Care Med..

[38] Butland R.J., Pang J., Gross E.R., Woodcock A.A., Geddes D.M. (1982). Two-, Six-, and 12-Minute Walking Tests in Respiratory Disease. Br. Med. J. (Clin. Res. Ed.).

[39] Ditunno J.F., Ditunno P.L., Graziani V., Scivoletto G., Bernardi M., Castellano V., Marchetti M., Barbeau H., Frankel H.L., D’Andrea Greve J.M. (2000). Walking Index for Spinal Cord Injury (WISCI): An International Multicenter Validity and Reliability Study. Spinal Cord.

[40] Dittuno P.L., Ditunno J.F., Dittuno J.F. (2001). Walking Index for Spinal Cord Injury (WISCI II): Scale Revision. Spinal Cord.

[41] Burns A.S., Delparte J.J., Patrick M., Marino R.J., Ditunno J.F. (2011). The Reproducibility and Convergent Validity of the Walking Index for Spinal Cord Injury (WISCI) in Chronic Spinal Cord Injury. Neurorehabil. Neural Repair.

[42] Perry J., Garrett M., Gronley J.K., Mulroy S.J. (1995). Classification of Walking Handicap in the Stroke Population. Stroke.

[43] Catz A., Itzkovich M., Agranov E., Ring H., Tamir A. (1997). SCIM--Spinal Cord Independence Measure: A New Disability Scale for Patients with Spinal Cord Lesions. Spinal Cord.

[44] Catz A., Itzkovich M., Steinberg F., Philo O., Ring H., Ronen J., Spasser R., Gepstein R., Tamir A. (2001). The Catz-Itzkovich SCIM: A Revised Version of the Spinal Cord Independence Measure. Disabil. Rehabil..

[45] Catz A., Itzkovich M., Tesio L., Biering-Sorensen F., Weeks C., Laramee M.T., Craven B.C., Tonack M., Hitzig S.L., Glaser E. (2007). A Multicenter International Study on the Spinal Cord Independence Measure, Version III: Rasch Psychometric Validation. Spinal Cord.

[46] Banaszek G. (2010). Vojta’s method as the early neurodevelopmental diagnosis and therapy concept. Przegl Lek.

[47] Berg K. (1989). Measuring Balance in the Elderly: Preliminary Development of an Instrument. Physiother. Can..

[48] Inness E.L., Howe J.-A., Niechwiej-Szwedo E., Jaglal S.B., McIlroy W.E., Verrier M.C. (2011). Measuring Balance and Mobility after Traumatic Brain Injury: Validation of the Community Balance and Mobility Scale (CB&M). Physiother. Can..

[49] Forrest G.F., Hutchinson K., Lorenz D.J., Buehner J.J., Vanhiel L.R., Sisto S.A., Basso D.M. (2014). Are the 10 Meter and 6 Minute Walk Tests Redundant in Patients with Spinal Cord Injury?. PLoS ONE.

[50] Guyatt G.H., Pugsley S.O., Sullivan M.J., Thompson P.J., Berman L., Jones N.L., Fallen E.L., Taylor D.W. (1984). Effect of Encouragement on Walking Test Performance. Thorax.

[51] Musselman K.E., Yang J.F. (2014). Spinal Cord Injury Functional Ambulation Profile: A Preliminary Look at Responsiveness. Phys. Ther..

[52] Ball C., Sackett D., Phillips B., Haynes B., Straus S. Levels of Evidence and Grades of Recommendations. http://www.cebm.net/oxford-centre-evidence-based-medicine-levels-evidence-march-2009/.

